# Study on the relationship between PM_2.5_ concentration and intensive land use in Hebei Province based on a spatial regression model

**DOI:** 10.1371/journal.pone.0238547

**Published:** 2020-09-18

**Authors:** Jingjing Shao, Jingfeng Ge, Xiaomiao Feng, Chaoran Zhao

**Affiliations:** 1 College of Resources and Environment, Hebei Normal University, Shijiazhuang, Hebei Province, China; 2 Hebei Key Laboratory of Environmental Change and Ecological Construction, College of Resources and Environment, Hebei Normal University, Shijiazhuang, Hebei Province, China; 3 College of Resources and Environment, Shijiazhuang University, Shijiazhuang, Hebei Province, China; Institute for Advanced Sustainability Studies, GERMANY

## Abstract

Based on 0.01°×0.01° grid data of PM_2.5_ annual concentration and statistical yearbook data for 11 cities in Hebei Province from 2000 to 2015, the temporal and spatial distribution characteristics of PM_2.5_ in the study area are analysed, the level of intensive land use in the area is evaluated, and decoupling theory and spatial regression are used to discuss the relationship between PM_2.5_ concentration and intensive land use and the influence of intensive land use variables on PM_2.5_ in Hebei Province. The results show that 1. In terms of time, the concentration of PM_2.5_ in Hebei Province showed an overall upward trend from 2000 to 2015, with the highest in winter and the lowest in summer. The daily variations show double peaks at 8:00–10:00 and 21:00–0:00 and a single valley at 16:00–18:00. 2. In terms of space, the concentration of PM_2.5_ in Hebei Province is high in the southeast and low in the northwest, and the pollution spillover initially decreases and then increases. 3. In the past 16 years, the level of intensive land use in Hebei Province has increased annually, but blind expansion still exists. 4. Decoupling theory and the spatial lag model show that land use intensity, land input level and land use structure are positively correlated with PM_2.5_ concentration, land output benefit is negatively correlated with PM_2.5_ concentration, and PM_2.5_ concentration and land intensive use level have not yet been decoupled; thus, the relationship is not harmonious. This research can provide a scientific basis for reducing air pollution and promoting the development of urban land resources for intensive and sustainable development.

## Introduction

After 1978, the area of urban land in China expanded rapidly and the urbanization rate increased rapidly. The urbanization rate exceeded 56% in 2015 [1, 2], and it is expected to reach 60% by 2020 [3]. Land is an important carrier of economic activities and a rare and non-renewable resource. In the Beijing-Tianjin-Hebei area, which has high urbanization levels, land resources available for development are very rare. Therefore, making intensive use of land resources and building compact cities have become important issues for the development of Hebei Province [4, 5].

However, with the development of urban "compactness", land use and human activities are increasingly concentrated in smaller spaces and environmental problems, especially air pollution characterized by haze, are particularly prominent. On the basis of the new air-quality criteria in 2012, the average air quality compliance rate in Hebei Province was 35.3%. In 2013–2018, Hebei Province had the worst air quality in China. PM_2.5_ (particulate matter less than 2.5 μm) is the primary air pollutant in Hebei Province and the most important "culprit" of haze weather [6]. Compared with other air pollutants, PM_2.5_ can more easily absorb all kinds of toxic and harmful substances and cause respiratory or cardio-cerebrovascular diseases [7, 8]. In addition, high concentrations of PM_2.5_ results in a decrease in atmospheric visibility, makes traffic accidents more frequent and brings great inconvenience to people's travel and transportation [9].

Now, PM_2.5_ research mainly focuses on the element composition [10], source analyses [11], health effects [12], management and impact factors [13], etc. The research mostly adopts qualitative or simple quantitative analysis from the macro perspective, and the commonly used analysis methods are artificial neural network [14], land use regression analyses [15] and spatial interpolation simulation [16]. Studies show that PM_2.5_ mainly comes from a large amount of industrial waste gas, automobile exhaust emissions and emissions from other activities [17–19], presents significant spatial-temporal distribution characteristics [20], and is affected by climate and meteorological conditions [21], urban economic development and other social factors [22]. These studies greatly improve people's cognition of PM_2.5_ pollution at the scientific level. In recent years, research on the factors influencing pollutants has gradually deepened. Some scholars take into account land use related variables, such as urban construction land, green area, road density, etc. [23]. However, the impact of land intensive use variables on PM_2.5_ does not an in-depth analysis.

Therefore, in view of the deficiencies of current studies and the urgency of haze control in Hebei Province. In this paper, PM_2.5_ concentration is calculated by using 0.01°×0.01° grid data of PM_2.5_ annual concentration. Land use intensity, land use structure, land input level and land output benefit are selected as the variables of intensive land use, and a decoupling analysis, spatial autocorrelation analysis, spatial regression model are adopted to comprehensively analyse the effects of intensive land use variables on PM_2.5_. The results are of great significance for improving intensive land use, enhancing air quality and promoting coordinated development.

## Material and methods

### Study area

**Fig 1:**
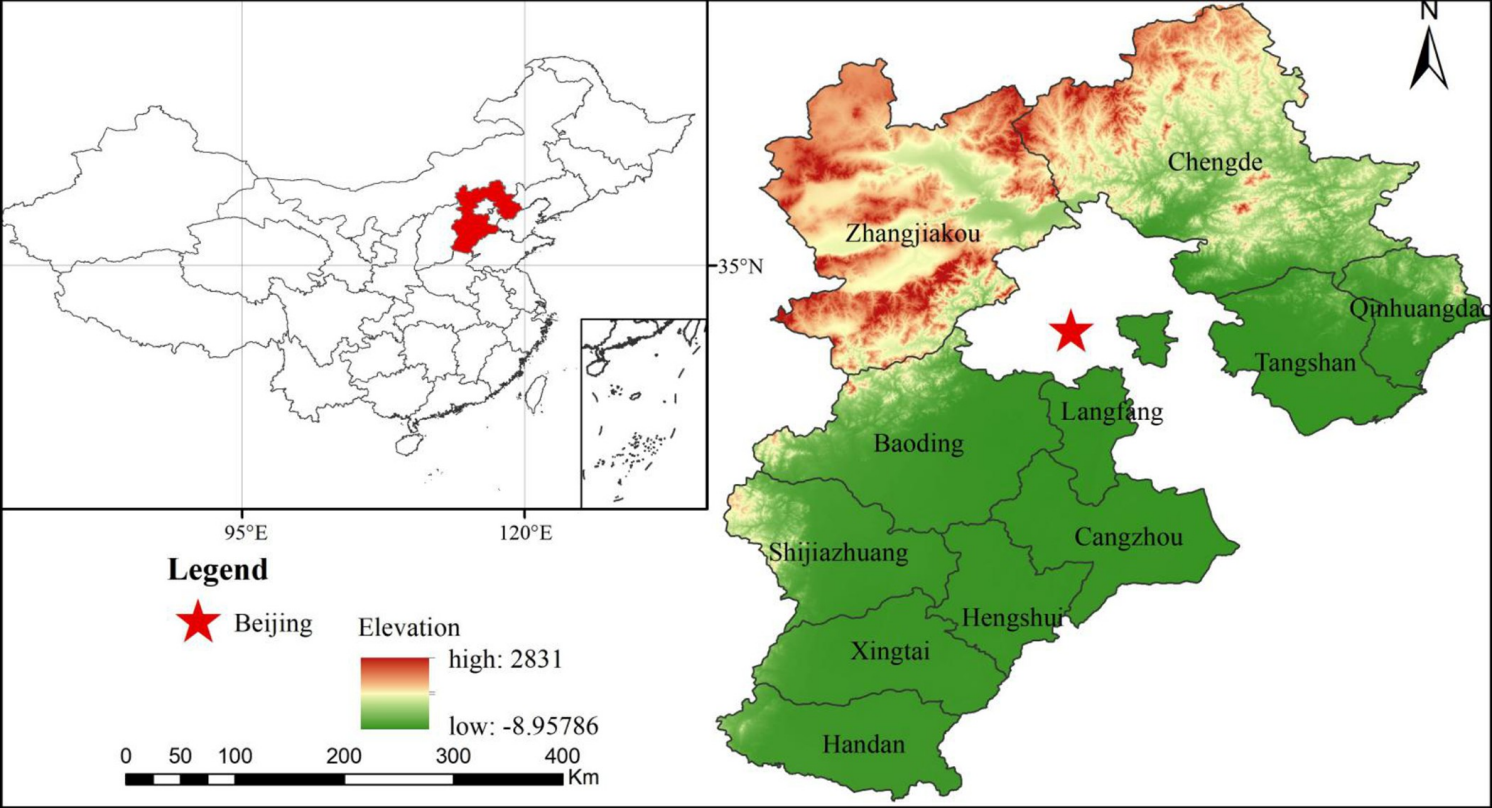
Location map of study area.

Hebei Province is situated between 37°57′N-38°12′N, 113°04′E-114°45′E and bordered by Beijing and Tianjin (Fig 1). Its total area is 188600 square kilometres. The landform is complex and diverse, with complete types. The province has a temperate continental monsoon climate, with rain and heat over the same period, and four distinct seasons. Hebei Province contains 11 cities, and the capital is Shijiazhuang. With the introduction of “Planning outline for coordinated development of Beijing, Tianjin and Hebei”, the economy of Hebei Province has continued to grow rapidly. In 2018, Hebei's GDP reached 3601.03 billion yuan, with an economic growth rate of 6.6%, thus ranking ninth among China's 31 provinces.

### Data

In this paper, the PM_2.5_ concentration grid data (0.01°×0.01°) in 2000, 2005, 2010, 2015 and the hourly PM_2.5_ concentration data in 2015 are obtained from the Atmospheric Composition Analysis Group (http://fizz.phys.dal.ca/~atmos/martin/?page_id=140). In addition, the data needed for intensive land use evaluations include construction land area, population density, urbanization rate, park green area, road land area, cultivated land area, financial expenditure, total number of employees in the second and third industries, total fixed asset investment, output value of the second and third industries, total retail sales of consumer goods, green coverage rate of the built-up area, total population and built-up area. These data are from the Hebei statistical yearbook and China urban statistical yearbook in 2000, 2005, 2010 and 2015.

### Methods

#### Gravity model

With the acceleration of the process of "regional economic integration", the distance between regions is shrinking; moreover, because of the natural attribute of long-distance diffusion of air pollution, it is impossible for air pollution treatments in different regions to be independent and not to affect each other [24, 25]. A large number of studies show that the spatial distribution of PM_2.5_ pollution in Hebei Province presents very obvious correlation, agglomeration and association characteristics [26–28]. The gravity model is one of the most important function forms to describe the spatial distribution association of regions [29, 30]. Compared with the VAR (Vector Autoregressive) model, the gravity model is more suitable for aggregate data, and it also considers the influence of economic geographical distance [31]. On this basis, Liu constructed a gravity model of pollution spillover. According to the work of Liu, this research uses the PM_2.5_ annual concentration as a measurement index to improve the gravity model.

$$y_{ij}=k_{ij}\frac{\sqrt[{3}]{P_{i}E_{i}G_{i}}\sqrt[{3}]{P_{j}E_{j}G_{j}}}{{\left(D_{ij}/\left(g_{i}‐g_{j}\right)\right)}^{2}},k_{ij}=\frac{E_{i}}{E_{i}+E_{j}}$$ (1)

Where *i* and *j* represent cities; *y*_*ij*_ is the gravity of air pollutants between city *i* and city *j*; *E*_*i*_ and *Ej* are the annual average PM_2.5_ concentrations of city *i* and city *j*, respectively; *Pi* and *Pj* are the total population of city *i* and city *j* at the end of the year, respectively; *G*_*i*_ and *G*_*j*_ are the actual GDP of city *i* and city *j*, respectively; *kij* represents the contribution rate of city *i* in the spatial association of air pollutants between city *i* and city *j;* Considering the influence of economic distance and geographical distance on the spatial association of PM_2.5_, this study applies the ratio of the distance (*Dij*) between the government locations of city *i* and city *j* to the difference between the per capita GDP of city *i* and city *j* (*gi*-*gj*) to express the distance. According to formula 1, the gravity matrix of air pollutant emissions between cities is calculated.

#### Construction of an intensive land use evaluation system

Considering previous research results, the principles of data availability and scientific rigor as well as the actual situation of Hebei Province, this paper selects 14 indicators in the four major directions of land use intensity to evaluate intensive land use in Hebei Province (Table 1).

**Table 1:** Evaluation index system of intensive land use.

Target Layer	Criteria Layer	Index Layer	Number	Units	Properties
Urban Land Intensive Utilisation Level	Land use intensity (X1)	Per capita construction land area	x1	m^2^/Person	-
		Population density	x2	Person/km^2^	+
		Urbanisation rate	x3	%	-
	Land use structure (X2)	Proportion of green area	x4	%	+
		Proportion of road area	x5	%	+
		Proportion of cultivated land	x6	%	+
	Land input level (X3)	Fiscal expenditure per square kilometre of land	x7	Ten thousand yuan/km^2^	+
		Proportion of employees in secondary and tertiary industries	x8	%	+
		Fixed asset investment per square kilometre of land	x9	Ten thousand yuan/km^2^	+
	Land output benefit (X4)	Per capita GDP(PGDP)	x10	Yuan/Person	+
		Output value of the secondary and tertiary industries per square kilometre of land	x11	Ten thousand yuan/km^2^	+
		Total retail sales of consumer goods per square kilometre of land	x12	Ten thousand yuan/km^2^	+
		Green coverage rate of built-up area	x13	%	+
		Per capita green area	x14	%	+

The evaluation of intensive land use usually involves several indicators, and setting the weight of each indicator has been one of the controversial points in past research. The analytic hierarchy process [32], data envelopment model [33], principal component analysis [34] and other methods have been commonly used in past research. However, these methods inevitably have certain subjectivity [35]. To prevent the subjective setting of index weights from affecting the objectivity of the research results, this study adopts the entropy method to objectively evaluate the weight of indexes. Afterwards, the scores of four criteria layers are calculated for each city to determine the intensive land use level of the city.

Because of the inconsistency of each index unit, the initial matrix needs to be standardized in data processing. In this paper, each index value adopts the difference method for the standardization of treatment. The original data matrix **X** = (*x*_*ij*_) is composed of 11 cities and 14 evaluation indexes of Hebei Province in 2000, 2005, 2010 and 2015, where *i* = 1,2,3…*n*; *j* = 1,2,3…*m*; *x*_*ij*_ is the *j*th index value of city *i*.

$$\mathrm{Positive}\;\mathrm{index}:\;S_{ij}=\frac{x_{ij}‐\min(x_{ij})}{\max(x_{ij})‐\min(x_{ij})}$$ (2)

$$\mathrm{Negative}\;\mathrm{index}:\;S_{ij}=\frac{\max(x_{ij})‐x_{ij}}{\max(x_{ij})‐\min(x_{ij})}$$ (3)

In formulas 2–3, *S*_*ij*_ means the result after standardization, *x*_*ij*_ means the original value, min(*x*_*ij*_) means the minimum value of *x*_*ij*_, and max(*x*_*ij*_) means the maximum value of *x*_*ij*_. The values are between [0,1].

After all the data are standardized, and each index is given a weight by the entropy method. The specific method is as follows:

First, calculate the proportion of city *i* under index *j*.

$$P_{ij}=\frac{S_{ij}}{\sum_{i=1}^{n}S_{ij}}$$ (4)

Then, calculate the entropy value *e*_*j*_ and the difference coefficient *gj* of index *j*.

$$e_{j}=‐k\sum_{i=1}^{n}P_{ij}\mathrm{In}(P_{ij})$$ (5)

k=1/In(n) (6)

$$g_{j}=1‐e_{j}$$ (7)

Finally, the weight of each index is obtained by formula 8.

$$W_{j}=\frac{g_{j}}{\sum_{j=1}^{m}g_{j}}$$ (8)

According to the constructed evaluation system of intensive land use, scores of sub goals and overall goals of each city in Hebei Province in 2000, 2005, 2010 and 2015 are calculated by formula 9 and formula 10, respectively.

$$B_{ib}=\sum_{j=1}^{m}W_{j}S_{ij}(i=1,2,\cdots ,n;b=1,2,3,4)$$ (9)

$$A_{i}=\sum_{b=1}^{4}B_{ib}(i=1,2,\cdots ,n;b=1,2,3,4)$$ (10)

In formulas 9–10, *Sij* means the standardized data of city *i* under index *j*, *Wj* is the weight of index *j*, and B*ib* is the score of sub goal *b* of city *i*. Add the scores of each sub goal using formula 10 to obtain the scores of each city's total goals. To facilitate the comparison, the score needs to be expressed as a centesimal system. The results are expressed in *Ci*, and the formula is as follows:

$$C_{i}=40A_{i}+60$$ (11)

#### Spatial autocorrelation analysis

The second-order effect of data generated by the similarity of variables in adjacent regions is called spatial autocorrelation, which is usually measured by Moran's I index [36] or Geary's C index [37]. In this paper, Moran's I index is adopted.

$$I=\frac{n\sum_{i=1}^{n}\sum_{j=1}^{n}W_{ij}(x_{i}‐\bar{x})(x_{j}‐\bar{x})}{(\sum_{i=1}^{n}\sum_{j=1}^{n}W_{ij})\sum_{i=1}^{n}{\left(x_{i}‐\bar{x}\right)}^{2}}=\frac{\sum_{i=1}^{n}\sum_{j=1}^{n}W_{ij}(x_{i}‐\bar{x})(x_{j}‐\bar{x})}{(\sum_{i=1}^{n}\sum_{j=1}^{n}W_{ij})s^{2}}$$ (12)

where *n* is the total number of cities, *wij* is the row standardized spatial weight matrix, *xi* and *xj* are the PM_2.5_ observation values of city *i* and city *j*, respectively, $\bar{x}=\frac{1}{n}\sum_{i=1}^{n}x_{i}$ indicates the average values of the PM_2.5_ concentrations of all cities and $s^{2}=\frac{1}{n}\sum_{i=1}^{n}(x_{i}‐\bar{x}{)}^{2}$ indicates the variance of the observation values. The *I* value is from [–1, 1]. When the *I* value is greater than 0, it indicates positive spatial autocorrelation. The higher *I* value, the stronger aggregation degree; that is, high value areas are adjacent to each other, and low value areas are adjacent to other low value areas. When the *I* value is less than 0, it indicates negative spatial autocorrelation; that is, high value areas are adjacent to low value areas.

#### Spatial regression model

The linear regression model is a classical model in econometrics, although when there is spatial correlation and spatial heterogeneity between variables, the results present serious deviation, which can affect the reliability of the research conclusions. Spatial econometrics can effectively solve the problem of spatial correlation between variables by applying classical statistical and econometric methods to geospatial data related to geographical location and spatial interaction. By establishing a relationship between geographical location and statistical variables, the laws of spatial change and the determinants of spatial patterns can be identified [38, 39]. Spatial lag model (SLM), spatial error model (SEM) and spatial Durbin model (SDM) are widely adopted spatial econometric models.

The SLM is mainly used to research the impact of PM_2.5_ emissions from neighbouring regions to other regions (formula 13).

$$Y=\rho WY+X\beta +\varepsilon ,\varepsilon ∼N(0,\delta^{2})$$ (13)

Where *Y* is an *n*×1-dimensional dependent variable vector, representing the annual average PM_2.5_ concentration; *X* represents the explanatory variables (four independent variables are included in this paper); *WY* is a spatial lag dependent variable; *ρ* is a spatial regression coefficient that can reflect the spatial dependence of sample observations and reveal whether the dependent variable presents a diffusion phenomenon (spillover effect) in a region; *β* is a regression coefficient; *ε* is a random error term, which is generally considered to be randomly distributed.

The SEM is needed when the regional interaction differs according to the relative position. The model takes full account of the influence of spatial correlation error terms on dependent variables (formula 14).

$$Y=X\beta +\lambda Wu+\varepsilon ,\varepsilon ∼N(0,\delta^{2})$$ (14)

where *u* is the random error vector, *λ* is the spatial error coefficient of the *n*×1-order dependent variable vector, and *W* is the spatial weight matrix of *n*×*n* order.

The SDM is a more universal form of the SLM and the SEM. The SLM contains the endogenous interaction effect of the explained variable (*Y*), the SEM contains the interaction effect between error terms (*ε*), and the SDM contains the endogenous interaction effect (*WY*) and exogenous interaction effect (*WX*) [40].

$$Y=\rho WY+X\beta +WX\theta +\varepsilon ,\varepsilon ∼N(0,\delta^{2})$$ (15)

where *θ* is the coefficient of the exogenous interaction effect.

Moran's I, Wald, LM-lag, LM-error and robust LM-lag and LM-error tests can be adopted to choose which space panel model to use. First, the Moran's I test is adopted to examine whether the dependent variable has spatial autocorrelation. If results show spatial correlation, the Wald test, LM test and robust LM test are further adopted to select models. If the Wald test is passed, the SDM is selected. If not, the LM test and robust LM test are applied. If the LM-lag is more prominent than the LM-error, the robust LM-lag also passes the test, while the robust LM-error does not, then the SLM is selected. If the LM-error is more prominent than the LM-lag in statistical data, the robust LM-error passes the test, while the robust LM-lag fails the test, then the SEM is selected. In this paper, the spatial regression model is built in MATLAB 2016a.

#### Decoupling theory

Decoupling means that the relationship between two or more physical quantities with response relationship no longer exists [41]. Latterly, this theory has been introduced into geography. The term "decoupling" is used to express the interruption of the relationship between economic growth and resource consumption or environmental pollution. In other words, economic growth should be decoupled from environmental pollution to realize sustainable development [42]. Since the Organization of Economy Cooperation and Development (OECD) formally defined decoupling indicators in 2002 [43], decoupling indexes have been published for different countries every year. However, the decoupling indicators defined by the OECD are highly sensitive to the selection of base year, and different results arise under different base years; therefore, these indicators cannot accurately describe the state of decoupling [44]. To solve the uncertainty of base year selection, Tapio defines decoupling indicators and divides decoupling indicators into three types and eight states [45]. Based on Tapio decoupling theory, this study carried out the corresponding variable transformation and constructed the following formula:

$$DI=\frac{\Delta PM_{2.5}}{\Delta LUI}=\frac{\left(PM_{2.5}{}_{TE}‐PM_{2.5}{}_{TB}\right)/PM_{2.5}{}_{TB}}{(LUI_{TE}‐LUI_{TB})/LUI_{TB}}$$ (16)

*DI* is the decoupling index. Δ*PM*_2.5_ is the change rate of PM_2.5_; Δ*LUI* is the change rate of intensive land use intensity; and *TE* and *TB* are the end year and start year of period T, respectively. Following Tapio's research, the decoupling state be divided into 8 decoupling states, as shown in Table 2. In this paper, the decoupling of intensive land use and air pollution means that the increase in intensive land use will not lead to the increase in air pollution; that is, the strong decoupling state is the ideal mode.

**Table 2:** Tapio decoupling status division table.

Type	State	Change rate of PM_2.5_	Change rate of land intensity use	Decoupling index
Negative decoupling	Expansive negative decoupling	>0	>0	DI>1.2
	Strong negative decoupling	>0	<0	DI<0
	Weak negative decoupling	<0	<0	0<DI<0.8
Decoupling	Strong decoupling	>0	>0	DI<0
	Weak decoupling	<0	>0	0<DI<0.8
	Recessive decoupling	<0	<0	DI>1.2
Coupling	Expansive coupling	>0	>0	0.8<DI<1.2
	Recessive coupling	<0	<0	0.8<DI<1.2

## Results and discussion

### Temporal and spatial distribution characteristics of PM_2.5_

#### Temporal distribution characteristics

**Fig 2:**
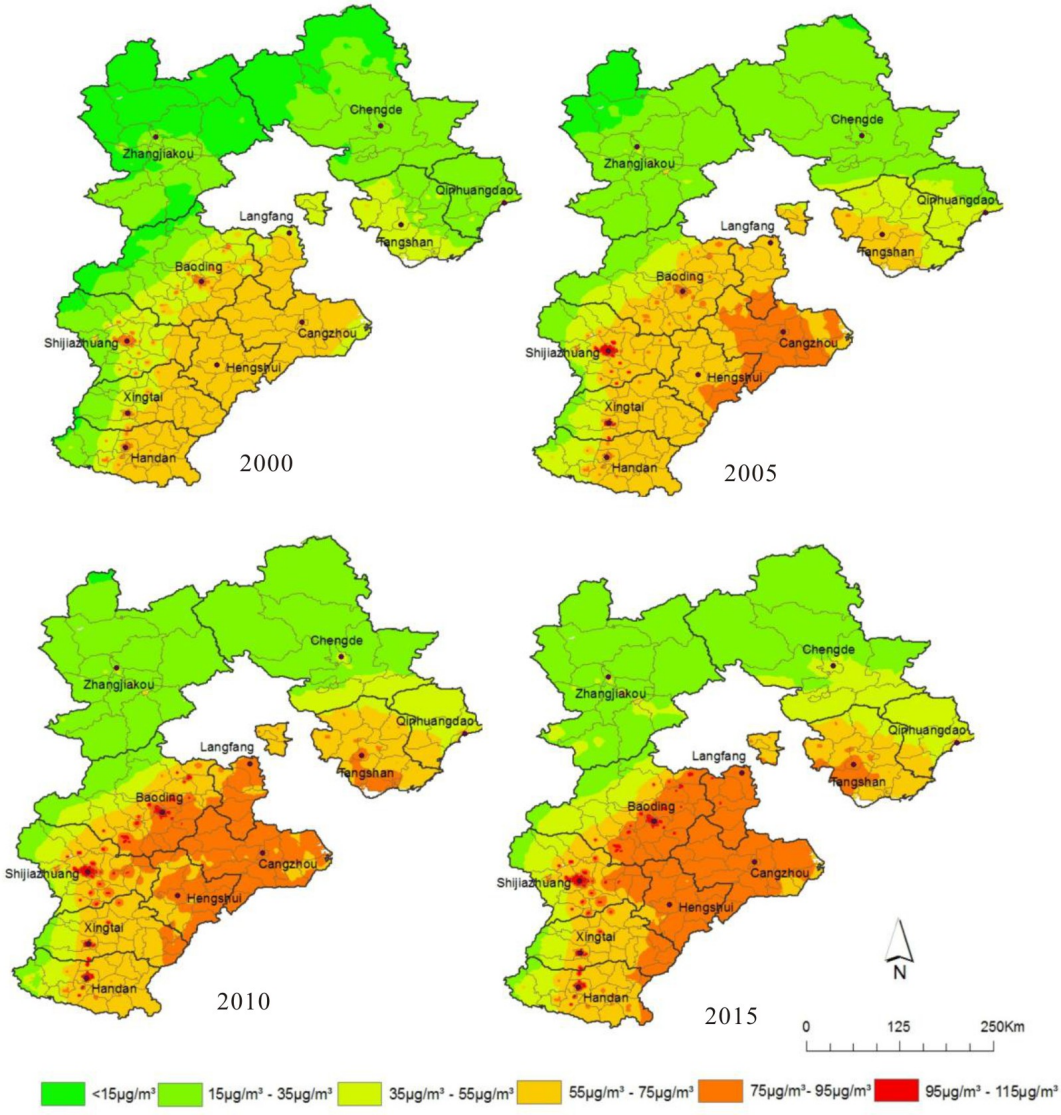
PM_2.5_ concentration distribution in Hebei Province from 2000 to 2015.

The problem of air pollution in Hebei Province has a long history, and it has experienced explosive growth in the 21st century. Fig 2 clearly displays that the PM_2.5_ concentration from 2000 to 2015 follows an increasingly high characteristic. In 2000, about half of the areas exceeded the annual average limit (35 μg/m^3^), and only in the areas near the urban built-up area reached more than 75 μg/m^3^. However, in 2015, approximately two-thirds of the study area was more than 35μg/m^3^, and Xingtai, Handan, Baoding, and Shijiazhuang was even more than 95 μg/m^3^, proving that the PM_2.5_ problem in Hebei Province was worsening.

**Fig 3:**
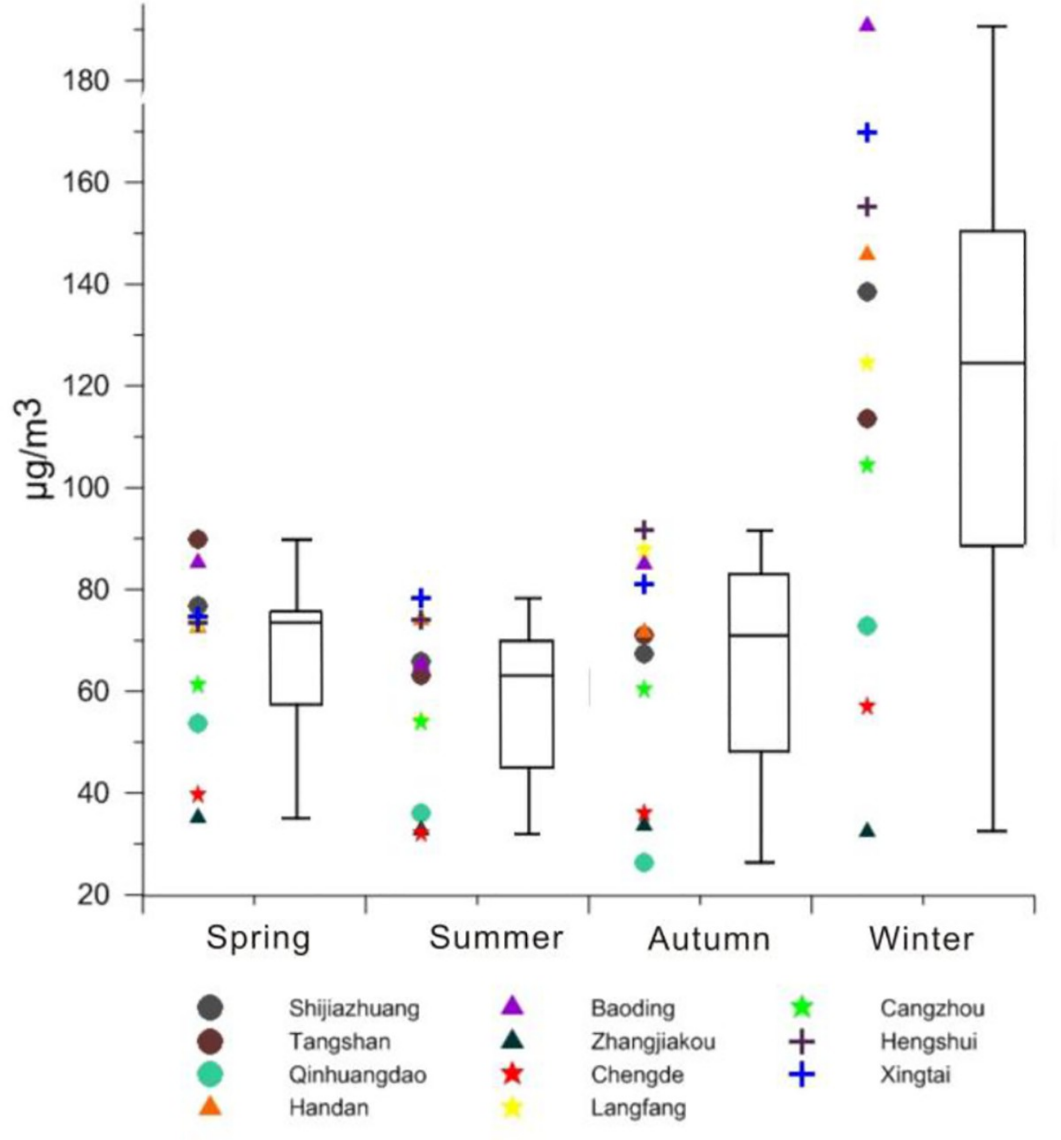
Box diagram of PM_2.5_ concentration in the four seasons of Hebei Province in 2015.

According to the hourly PM_2.5_ concentration data of Hebei Province in 2015, the monthly average value in each city is calculated. On this basis, a box diagram in the four seasons is drawn for Hebei Province (Fig 3). The average concentration is the maximum in winter, reaching 118.57 μg/m^3^, followed by 66.87 μg/m^3^, 64.70 μg/m^3^ and 57.23 μg/m^3^ in spring, autumn and summer. It can be seen from the long box length in winter that the dispersion degree of particle concentration is very high, which indicates that extreme pollution events often occur during this period. This is related to the fact that the thermal energy companies in Hebei Province need to consume more fossil fuels when entering the heating period in winter. Moreover, the atmosphere in winter is relatively stable, and the frequency of temperature inversion are high; such climate conditions are not conducive to the dilution and diffusion of pollutants. Furthermore, on the Spring Festival, the concentration of PM_2.5_ increases due to the concentrated discharge of a large amount of fireworks. In spring and autumn, the climate is dry, windy and rainless, with dust raising conditions, and straw burning occurs in autumn; therefore these seasons rank higher than summer. In summer, the higher temperature, decreased atmospheric stability and concentrated rainfall are helpful to the diffusion, deposition and dilution of PM_2.5_. Therefore, the lowest ranking is summer.

**Fig 4:**
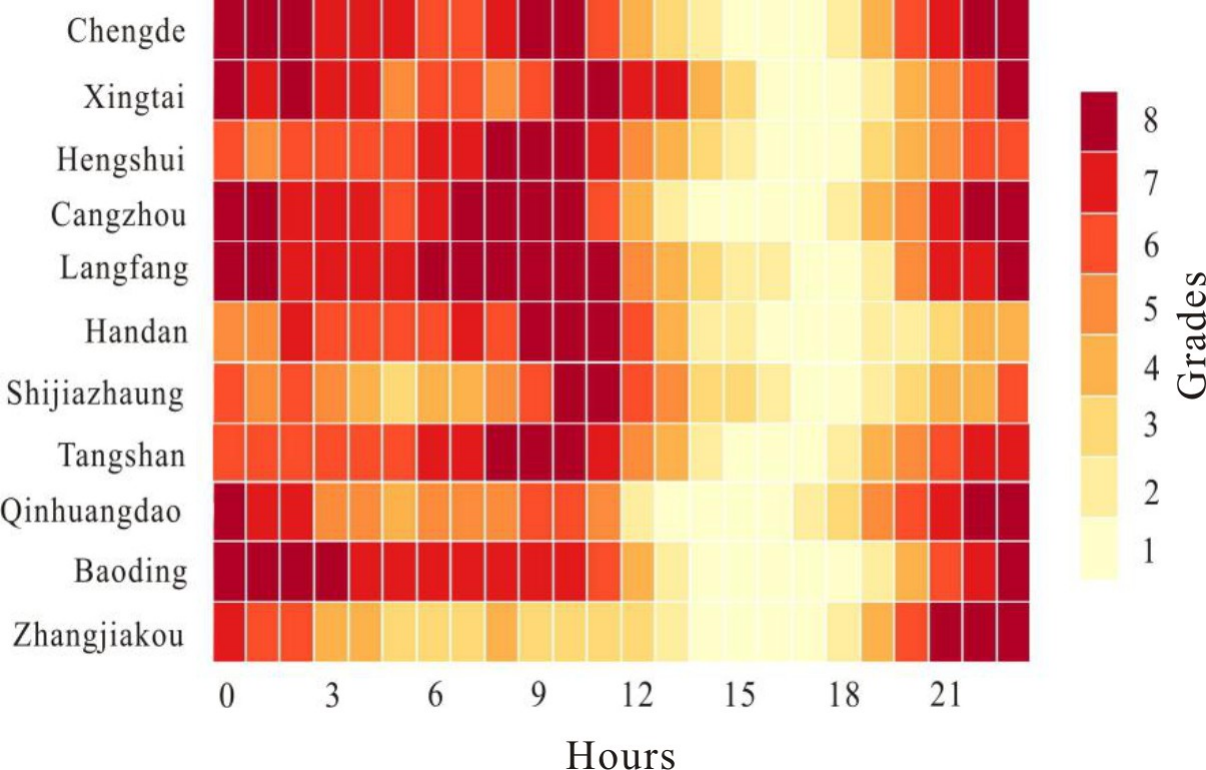
Calendar chart of daily PM_2.5_ concentration in Hebei Province in 2015.

To further understand the time distribution rule of PM_2.5_ concentration in Hebei Province, the average hourly PM_2.5_ concentration in different cities in 2015 is calculated (Fig 4). On the whole, the change in the PM_2.5_ hourly mean value of each city in Hebei Province shows the phenomenon of double peaks and a single valley. The double peaks appear at 8:00–10:00 in the daytime and 21:00–0:00 at night. A single valley appears from 16:00 to 18:00 in the evening. A large number of studies show that the main sources of PM_2.5_ in cities are secondary particles, mobile sources and dust [46]. During the early peak period, motor vehicle emissions, gaseous sulfur dioxide, nitrogen oxides, fine particles and road dust are continuously input into the atmospheric environment. With the rise of the air temperature after sunrise, secondary particles begin to form, eventually making the peak concentration of particles appear around 8:00–10:00, and with the enhancement of the vertical exchange capacity of turbulence, the concentration of particles decreases. At night, due to surface radiation, the temperature of the air near the ground is lower than that of the air far above the ground, which leads to temperature inversion. The continuous thickening of the inversion layer leads to the accumulation of particulate matter and the air pollutants emitted by motor vehicles in the evening peak, which makes the concentration of PM_2.5_ increase again.

#### Spatial distribution characteristics

Fig 2 shows that the PM_2.5_ concentration in Chengde, Zhangjiakou, northern Qinhuangdao is relatively low, while that in Baoding, Shijiazhuang, Xingtai and Handan is relatively high, presenting the trend of a southeast high and northwest low.

With the implementation of engineering projects (such as the west-to-east natural gas transmission project and the west-east electricity transmission project) aimed at promoting the cross-regional flow of energy, the spatial association of PM_2.5_ emissions in various cities has become more universal and extensive, surpassing the "close neighbour" relationship in the simple geographical sense and gradually presenting a multi-threaded complex network structure. Based on the characteristics, this paper uses the modified gravity model to analyse the spatial association of PM_2.5_ and uses social network analysis (SNA) to re-examine the network structure of PM_2.5_ in Hebei Province.

**Fig 5:**
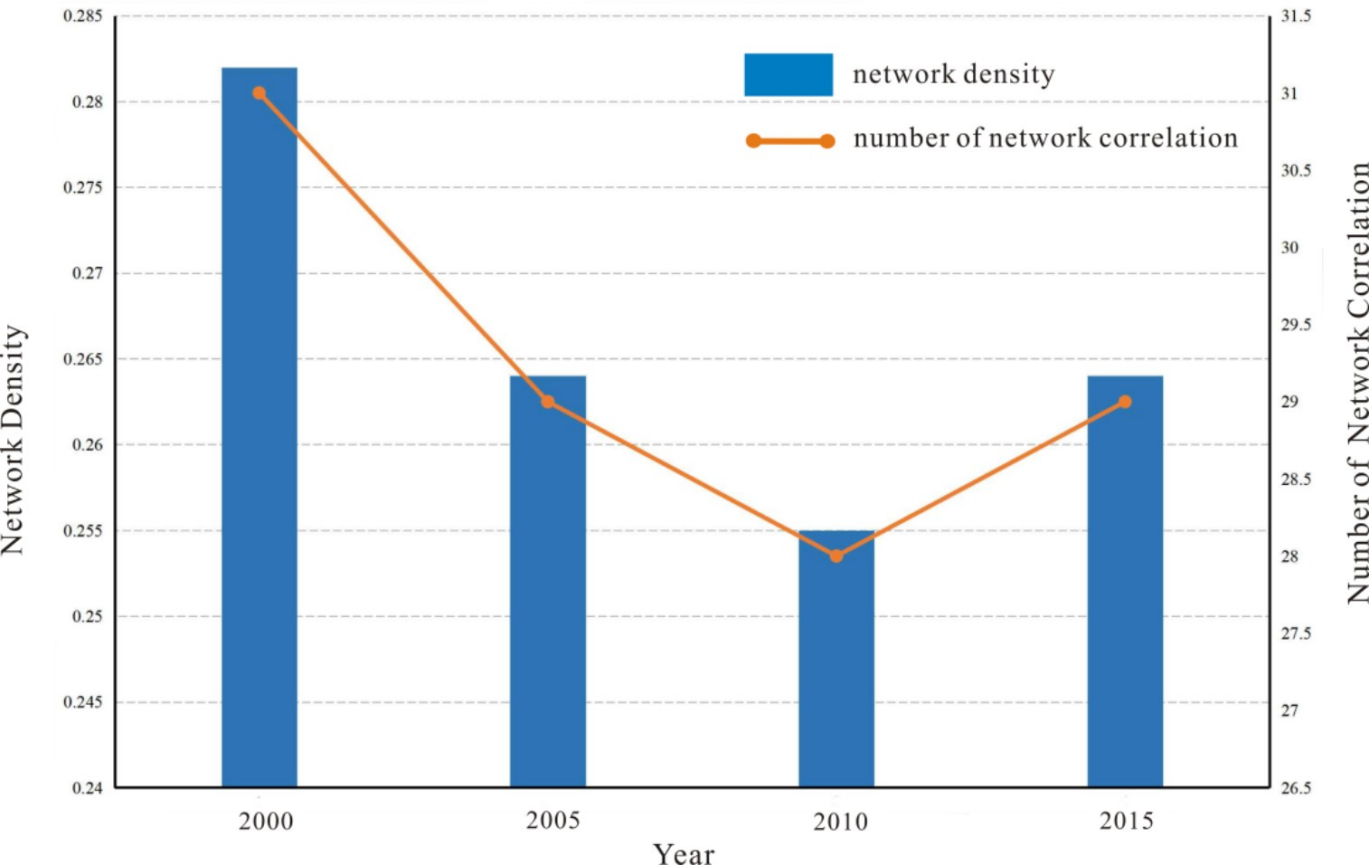
PM_2.5_ network density and number of network association from 2000 to 2015.

As a commonly used index in SNA, network density can reflect the degree of compactness of the PM_2.5_ spatial association network. With the increase of network density, the PM_2.5_ connection between cities also increases. The density of the whole network can be calculated using UCINET software, and the specific results are shown in Fig 5. This study can be divided into two periods. In the first period (2000–2010), the density of the PM_2.5_ spatial association network in Hebei Province decreased from 0.282 to 0.255. In these 11 years, the density of the PM_2.5_ spatial association network decreased by 9.57% and the average annual growth rate decreased by 0.87%, which shows that the PM_2.5_ pollution spillover phenomenon is weakening, and the change range is relatively small. The reason may be the increase in the number of days with stationary weather at this stage. To explore whether this result is related to stationary weather, the stationary weather index is introduced for analysis. The stationary weather index is a comprehensive meteorological condition index used to represent the diffusion of air pollutants. The larger the index is, the more favourable the weather conditions are for the generation and concentration of atmospheric pollution [47]. Zhao found that the correlation between the stationary weather index and the average PM_2.5_ concentration in Hebei is very good; that is, as the stationary weather index gradually increases (decreases), the PM_2.5_ concentration gradually increases (decreases), and the atmospheric diffusion conditions significantly deteriorate (improve) [48]. In the first period, the mean concentration of PM_2.5_ in Hebei Province gradually increased from 41.82 μg/m^3^ to 58.03 μg/m^3^. According to the conclusion that the stationary weather index is positively related to PM_2.5_, the stationary weather index value gradually increased in this stage. therefore, the air pollution spillover among cities in Hebei Province weakened. In the second period (2010–2015), the network density of the PM_2.5_ spatial association in Hebei Province increased to 0.264, with an average annual growth rate of 0.59%. The reason for this phenomenon may be that with the accelerated integration of informatization and industrialization at this stage, the social and economic development level of cities in Hebei Province is increasing at a high speed, and the total industrial output value and amount of pollution are correspondingly increasing, leading to increasing interregional pollution spillover year by year. In summary, this result reflects that the air pollution spillover in cities of Hebei Province is related to the weather, the economy and other natural economic factors.

### Evaluation of intensive land use in Hebei Province

Based on the statistical data of each index, the entropy weight method is used to compute the weight of index layers and criteria layers (Table 3).

**Table 3:** Weight table of evaluation indexes of study area in 2000, 2005, 2010 and 2015.

Target Layer	Criteria Layer	Index Layer	Mean Weight	2000	2005	2010	2015
Urban Land Intensive Utilisation Level	Land use intensity (X1)	Per capita construction land area	0.038	0.031	0.04	0.038	0.043
		Population density	0.051	0.048	0.049	0.051	0.055
		Urbanisation rate	0.063	0.089	0.046	0.046	0.071
	Land use structure (X2)	Proportion of green area	0.100	0.107	0.098	0.101	0.092
		Proportion of road area	0.076	0.072	0.067	0.085	0.079
		Proportion of cultivated land	0.051	0.046	0.050	0.053	0.053
	Land input level (X3)	Fiscal expenditure per square kilometre of land	0.058	0.050	0.059	0.060	0.062
		Proportion of employees in secondary and tertiary industries	0.032	0.030	0.032	0.034	0.033
		Fixed asset investment per square kilometre of land	0.078	0.080	0.067	0.081	0.085
	Land output benefit (X4)	Per Capita GDP(PGDP)	0.108	0.082	0.119	0.123	0.109
		Output value of the secondary and tertiary industries per square kilometre of land	0.096	0.097	0.12	0.083	0.084
		Total retail sales of consumer goods per square kilometre of land	0.063	0.060	0.062	0.065	0.066
		Green coverage rate of built-up area	0.078	0.084	0.101	0.060	0.067
		Per capita green area	0.109	0.125	0.093	0.116	0.101

From Table 3, the weights of the four criteria layers are 0.152, 0.226, 0.168 and 0.454. The land output benefit has a greater impact on intensive land use in Hebei Province, while land use intensity and land input level have a smaller impact and the impact of land use structure is average. According to the weight of a single index, the weights of per capita green area, per capita GDP, proportion of green land use, and the output value of the secondary and tertiary industries per square kilometre of land are all at a higher level, indicating a higher effect on the level of intensive land use in Hebei Province. The weights of the proportion of employees in secondary and tertiary industries, per capita construction land area and other indicators have a smaller effect on the level of intensive land use.

**Fig 6:**
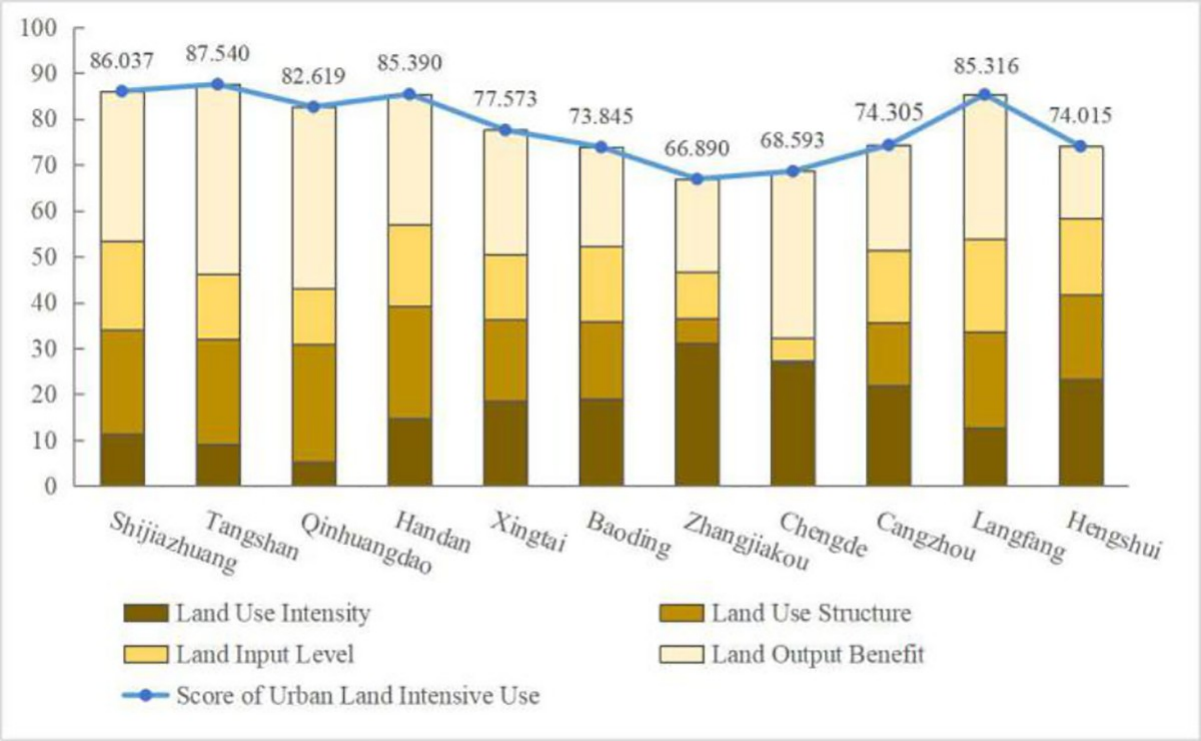
Chart of intensive land use and criterion layers average scores of cities in Hebei Province from 2000 to 2015.

**Fig 7:**
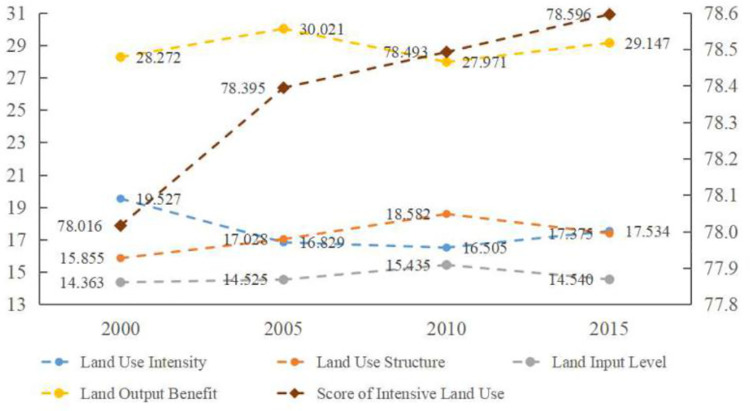
Chart of intensive land use and criterion layers evaluation results in Hebei Province from 2000 to 2015.

According to formulas 9–11, this paper obtains the comprehensive scores of intensive land use and the scores of each criterion layer for each city in Hebei Province in 2000, 2005, 2010 and 2015. According to the restriction of word number, this paper lists only the average scores of intensive land use for 11 cities in Hebei Province and the scores for each criterion layer in 2000, 2005, 2010, 2015, as reflected in Figs 6 and 7. As shown in Figs 6 and 7, from 2000 to 2015, the level of intensive urban land use showed a steady upward trend, among which Tangshan and Shijiazhuang ranked the highest and their economic development presented fast. Chengde and Zhangjiakou ranked the lowest, and their economic development presented slow. The score of Tangshan is 1.31 times that of Zhangjiakou. This shows that the level of intensive land use differs across the 11 cities in Hebei Province and has a positive correlation with the level of economic development. Based on the criterion level, the land output benefit, land use structure and land input level show a fluctuating upward trend while the land use intensity shows a declining phenomenon. This result shows that in recent years, the economic level of Hebei Province has gradually improved, the land use structure has become more reasonable and the investment in urban greening has gradually increased, which has led to an increase in the scores of the three criteria layers. However, although the land use intensity increased in 2010–2015, it mainly decreased compared with 2000 because in Baoding, Zhangjiakou and Cangzhou, which experienced rapid economic and social development, the construction land area expanded rapidly but the population growth lagged behind, resulting in the decrease in per capita construction land area, urbanisation rate and land use intensity.

### Relationship between PM_2.5_ and intensive land use in Hebei Province

#### Decoupling analysis

Combined with the characteristics of the Tapio decoupling analysis method, the research period is divided into three periods: T1 (2000–2005), T2 (2005–2010) and T3 (2010–2015). The PM_2.5_ change rate, land intensity use change rate and decoupling index of each city in Hebei Province in each period are computed, and the corresponding decoupling status is divided according to different decoupling indexes (Table 4).

**Table 4:** Decoupled status of PM_2.5_ and intensive land use level in cities of Hebei Province.

City	T1	T2	T3
Shijiazhuang	Strong negative decoupling	Expansive negative decoupling	Weak decoupling
	-2.531	1.929	0.359
Tangshan	Expansive negative decoupling	Strong negative decoupling	Weak negative decoupling
	1.621	-2.379	0.054
Qinhuangdao	Strong negative decoupling	Strong negative decoupling	Strong negative decoupling
	-3.670	-4.437	-0.092
Handan	Expansive negative decoupling	Expansive coupling	Strong negative decoupling
	1.608	0.979	-1.019
Xingtai	Strong negative decoupling	Strong negative decoupling	Strong negative decoupling
	-1.144	-1.067	-0.234
Baoding	Expansive negative decoupling	Expansive negative decoupling	Strong negative decoupling
	5.584	1.719	-1.771
Zhangjiakou	Strong negative decoupling	Strong negative decoupling	Weak decoupling
	-6.401	-0.308	0.182
Chengde	Strong negative decoupling	Weak decoupling	Weak decoupling
	-1.387	0.317	0.382
Cangzhou	Strong negative decoupling	Weak decoupling	Expansive negative decoupling
	-2.357	0.069	2.263
Langfang	Weak decoupling	Strong negative decoupling	Expansive negative decoupling
	0.442	-3.392	2.806
Hengshui	Strong negative decoupling	Strong negative decoupling	Weak decoupling
	-0.955	-1.838	0.133
Hebei Province	Expansive negative decoupling	Expansive negative decoupling	Expansive negative decoupling
	12.427	18.808	9.064

Table 4 shows that there are five decoupling states in the 16 years, namely, strong negative decoupling, expansive negative decoupling, weak decoupling, weak negative decoupling and expansive coupling, which account for 47.2%, 27.8%, 19.4% and 2.8%, respectively. The total number of negative decoupling types accounted for 77.8% of the total number of statistical periods, and expansive negative decoupling is the main state observed, indicating that intensive land use and air pollutant emissions in Hebei Province are still not decoupled. In other words, the enhancement of land use intensity in Hebei Province also caused the increase in PM_2.5_ concentration, and the increase in PM_2.5_ concentration was larger than the increase in land use intensity.

On the spatial scale, the decoupling state of different cities differs slightly in the same period. The decoupling state of Langfang, Chengde, Zhangjiakou, Shijiazhuang and Hengshui experienced strong negative decoupling to weak decoupling, that is, a transition from a non-ideal state to an ideal state. This shows that the relationship between intensive land use and air pollutant emission improved year by year and the increase in air pollutant emission intensity was effectively controlled with the increase in intensive land use. Cangzhou, Baoding, Handan and Tangshan mainly experienced the transformation from expansive negative decoupling to strong negative decoupling. This change shows that the disharmonious relationship became increasingly serious. Qinhuangdao and Xingtai mainly showed strong negative decoupling, which may be due to the increase of air pollutant emissions year by year, but the decrease of intensive land use level year by year, indicating that they are in a very incompatible state. In the future, more effective policies and economic means are needed to control the emission of PM_2.5_ and strengthen the level of intensive land use.

#### Spatial autocorrelation analysis

Based on the PM_2.5_ annual concentration grid data in 2000, 2005, 2010 and 2015, the spatial weight matrix is constructed using Geoda software, and the global Moran's I value of the PM_2.5_ annual average concentration of each city in Hebei Province is calculated (Table 5).

**Table 5:** Global autocorrelation analysis results.

Year	I value	Z value	P value
2000	0.5733	2.8031	0.008
2005	0.5387	2.6959	0.012
2010	0.4217	2.2247	0.030
2015	0.4667	2.4412	0.018

Moran’s I values are all positive and pass the significance test at a level of 0.05, which means that the spatial distribution of PM_2.5_ in each city of Hebei Province presents a significant and positive autocorrelation. From 2000 to 2010, Moran's I gradually decreased, showing a decreased spatial dependence of PM_2.5_ agglomeration and showing a trend of decentralized development. After 2010, Moran's I indicated a trend of growth, showing that the spatial dependence of PM_2.5_ in Hebei Province began to improve, which may be due to the development of regional economic agglomerations and the gradual transfer of polluting industries among regions.

#### Spatial effect of intensive land use on PM_2.5_

The Moran's I analysis verified the obvious spatial autocorrelation of PM_2.5_ in the cities of Hebei Province. Therefore, the general Ordinary Least Square (OLS) method cannot be used to analyse the non-independent sample data because the data do not support the basic assumption of the linear regression model, and the results will produce serious errors. Hence, this study adopts a spatial regression model for analysis. The Hausman test, LR test, Wald test, LM Test and robust LM Test are used to determine which spatial regression model to implement. See Table 6 for the specific results. The results show that the Wald_spatial_lag (9.7389, P>0.001) and Wald_spatial_error (4.0402, P>0.001) tests accept the original hypothesis that the SDM can be reducible to the SLM or SEM at the significance level of 0.001. The LM test passes the significance test at the level of 0.001, while the robust LM test rejects the original hypothesis that there is no spatial lag at the significance level of 0.001 but accepts the original hypothesis that there is no spatial error. Therefore, it is more appropriate to use the SLM than the SEM, and the spatial interaction effect of PM_2.5_ is again confirmed. If the general regression model is used, the results may be biased or even invalid. In addition, the Hausman test indicates that the fixed panel effect model is suitable. The results of the LR test of time and spatial fixed effects show that both are significant. Therefore, this research establishes a SLM of spatiotemporal double fixed effects to analyse the spatial effect of intensive land use on PM_2.5_.

**Table 6:** Test results of model selection.

Test	Statistic value	P value
Hausman	119.2751	0.000
Wald_spatial_lag	9.7389	0.045
Wald_spatial_error	4.0402	0.4006
LM test no spatial lag	42.1979	0.000
LM test no spatial error	28.8790	0.000
Robust LM test no spatial lag	14.6762	0.000
Robust LM test no spatial lag	1.3573	0.244
LR_test joint significance spatial fixed effects	152.7254	0.000
LR_test joint significance time fixed effects	102.8333	0.000

The above analysis shows that the spatiotemporal double fixed effects of the SLM is the most suitable in this paper, although for comparison purposes, the estimation results for the spatial fixed effect, temporal fixed effect and spatiotemporal double fixed effect of the SEM are also given (Table 7).

**Table 7:** PM_2.5_ and spatial econometric results of intensive land use.

Variable	SEM			SLM		
	Temporal fixed	Spatial fixed	Spatiotemporal double fixed	Temporal fixed	Spatial fixed	Spatiotemporal double fixed
Land use intensity	234.596***	83.563***	82.450***	197.316***	79.497***	90.383***
Land use structure	61.219	45.026	44.743	63.501	67.276**	44.169*
Land input level	104.412	150.449***	187.623***	115.695	182.201***	189.558***
Land output benefit	-20.980	-15.240*	-21.271**	-19.681	-18.617**	-21.086**
R^2^	0.6081	0.8597	0.9896	0.7490	0.9870	0.9905
Adjusted R^2^	0.5917	0.2564	0.5326	0.6726	0.6240	0.5506
Log-Likelihood	-166.264	-107.710	-90.837	-162.758	-105.669	-89.931
W*dep.var	——	——	——	0.416***	0.812***	0.326**
Spat.aut	0.483***	0.866***	0.249*	——	——	——

Note

*, **, ***, respectively means that the test is passed at the significance level of 10%, 5%, and 1%;—means that the content is empty.

Table 7 shows that the spatial lag term W*dep.var and the spatial error term Spat.aut of the econometric model pass the significance level test at 10%, which also verifies the rationality of adopting the spatial econometric model. A comparison of the log likelihood of the six models shows that the SLM has the best explanatory power. In addition, the R^2^ and adjusted R^2^ show that the spatiotemporal double fixed model is better, which also proves the correctness of the abovementioned robust LM test. Therefore, this paper uses the SLM with spatiotemporal double fixed effects for analysis.

There is a positive correlation between PM_2.5_ and land use intensity and land input level at the 1% significant level in Hebei Province, and the correlation coefficient is relatively high. The reason may be that the increase in population, urbanization rate, capital and human investment can provide sufficient capital and labour guarantees for urban municipal construction, economic growth, and development of the secondary and tertiary industry. However, it can cause an increase in PM_2.5_ because of coal-fired pollution sources, industrial and construction pollution sources, traffic pollution sources and catering oil smoke pollution sources along with the economic and population growth. There is a positive correlation between the land use structure and PM_2.5_ at the 10% significant level in Hebei Province. However, compared with the other two positive correlation variables, the impact of land use structure on PM_2.5_ is not very significant. This may be because, although the PM_2.5_ generated by traffic pollution sources increases due to the increase in the proportion of urban roads, the increase in the proportion of urban green space and the decrease in the proportion of cultivated land have a certain mitigation effect on the increase in atmospheric PM_2.5._ Therefore, the influence coefficient of land use structure on PM2.5 is less than that of land use intensity and land input level. Finally, the impact of land output benefit on PM_2.5_ is negatively correlated at the significance level of 5%, which proves that the increase in the per capita GDP, the output value of the secondary and tertiary industries per square kilometre of land and the total retail sales of consumer goods per square kilometre of land promote the development of the urban economy, resulting in the increase of PM_2.5_. However, the increase in urban green coverage and green area can prevent PM_2.5_ from entering local areas and play a role in dust suppression. Moreover, PM_2.5_ can be reduced by covering the surface of the earth to enhance dust reduction. In addition, the presence of trees can reduce the wind speed and promote particle settlement, the leaf surface can absorb and capture PM_2.5_, and the plant surface can absorb and transfer PM_2.5_, resulting in a decrease in PM_2.5_. Because the weight of the green space index in this criterion layer is large, the result is negative, which is also in line with the actual situation. In summary, on the basis of sustained population and economic growth, increasing the green area and green coverage is a way to reduce PM_2.5_, improve the level of land use intensification, and promote the coordinated development of the economy and the environment.

Intensive land use is an inevitable choice for future urban development. The connotation of intensive land use includes three aspects: economy, society and environment. Therefore, in the process of the intensive and efficient development, it is not sufficient to increase the efficient use of the economy and society in space only by enhancing investments and the agglomeration scale; rather it is also necessary to focus on the efficient use of the urban environment. For example, urban construction land can be changed from the two-dimensional "spreading cake" to the three-dimensional "stacking block", and the remaining construction land and bare land can be changed into green space, which can effectively alleviate the problem of urban air pollution by increasing vegetation. Moreover, the pillar industries of economic growth in Hebei Province are mostly high pollution industries; therefore the development scale of industry and the construction industry in Hebei Province should be reduced and the industrial structure should be optimized and become more advanced and reasonable. Such changes can not only promote economic growth and improve the level of intensive land use in the study area but also reduce PM_2.5_ emissions. In addition, according to this study, air pollution has obvious characteristics of cross-regional transmission. Therefore, when formulating air pollution prevention and control measures, studies should focus on investigating the interaction between urban agglomerations and the surrounding areas, establishing and strengthening joint prevention and control mechanisms and cooperative governance among regions, establishing inter-regional ecological compensation mechanisms, innovate pollution supervision and accountability modes, and promoting environmental protection and environmental legislation in urban agglomeration planning for the treatment of environmental pollution.

In 2015, the Ministry of Environmental Protection took the lead in analysing the sources of atmospheric particulate matter in key cities of Beijing-Tianjin-Hebei. Among them, the primary sources of pollution are motor vehicles in Beijing, coal in Shijiazhuang, and dust in Tianjin [49]. The project traces the source of the pollution particles, which is conducive to the formulation of targeted prevention and control policies. However, this study has some deficiencies, such as incomplete chemical composition analyses, uncertain emission sources, and lack of consideration of the impact of social factors, such as intensive land use, on air quality [50]. This paper can represent a useful supplement for the government environmental protection department for analysing pollutant sources and provide decision-making reference for local governments. In addition, considering the spatial diffusion effect of air pollution, it is necessary to consider the impact of geospatial correlations when exploring the relationship between regional air pollutants and intensive land use. Compared with the land use regressions, correlation analyses and other methods used in previous studies, the spatial econometric model is an effective method for investigating spatial correlation [51]. However, there are some shortcomings in this study. First, this research mainly analyses the impact of criteria layers on PM_2.5_ in an intensive land use evaluation system and makes a brief analysis considering the index weight. However, due to the limited data volume, the mechanism of the impact of each index layer on PM_2.5_ needs further study. Second, although the analysis object is at the level of the city area, it can macroscopically analyse the impact of intensive urban land use on PM_2.5_ concentration and the relationship between them. However, for industrial areas, residential areas and commercial areas, the relationship between intensive land use and PM_2.5_ concentration may need further study at the micro level.

## Conclusion

This paper is based on 0.01°×0.01° grid data of PM_2.5_ annual concentration and statistical yearbook data in Hebei Province from 2000 to 2015. The spatial and temporal characteristics of PM_2.5_, the change of intensive land use, the impact of intensive land use on PM_2.5_ and the relationship between them are studied using the gravity model, entropy weight method, Tapio decoupling theory, spatial autocorrelation analysis and spatial econometric model. The conclusions of the study include the following four aspects.

From 2000 to 2015, the concentration of PM_2.5_ in Hebei Province showed an overall upward trend. In space, PM_2.5_ concentration is high in the southeast and low in the northwest. According to the gravity model and social network analysis, the PM_2.5_ pollution spillover in Hebei Province can be divided into two periods. In the first period (2000–2010), the network density shows a downward trend. In the second period (2010–2015), the network density has increased. After comprehensive analysis, it was found that PM_2.5_ pollution spillover not only has a certain relationship with economic development but can also be affected by natural factors. There is an obvious difference across seasons, and the order of pollution degree is winter>spring>autumn>summer. In addition, there are two peaks and one valley in terms of the daily variation. The double peaks appeared at 8:00–10:00 and 21:00–0:00, and the average value of PM_2.5_ is higher at night than during the day. The single valley appeared at 16:00–18:00 in the evening.

The level of intensive land use in Hebei Province increased from 2000 to 2015, but obvious internal differences are observed. The cities with higher scores of intensive land use are Tangshan and Shijiazhuang, and the cities with lower scores are Chengde and Zhangjiakou, and these findings shows a positive correlation with the level of economic development. According to the sub-objectives of the intensive land use evaluation system, the land output benefit, land use structure and land input level show a fluctuating upward trend, while the land use intensity presents a downward trend. These findings show that land use is becoming increasingly reasonable, although blind expansion is still observed. According to the Tapio decoupling theory, the relationship between intensive land use and PM_2.5_ concentration in cities of Hebei Province in the past 16 years presents five states: strong negative decoupling, expansive negative decoupling, weak decoupling, weak negative decoupling and growth linkage. Across the whole province, the state of decoupling has been in expansive negative decoupling, which shows that intensive land use and PM_2.5_ concentration in Hebei Province are still not decoupled; that is, the relationship is not harmonious.

The global Moran’s I index shows that there is a significant positive spatial autocorrelation of PM_2.5_ concentration in each city of Hebei Province, indicating that there is a correlation between the PM_2.5_ concentration of a given city and its neighbouring cities. Further using the spatial econometric model, it is found that the SLM with spatiotemporal double fixed effects has the best fitting effect for this study. The results show that land use intensity, land input level and land use structure are positively correlated with PM_2.5_ concentration, while the land output benefit is negatively correlated with PM_2.5_ concentration.

## Supporting information

S1 Table Data of intensive land use in Hebei Province. It includes the original data of 14 land intensive use evaluation index of 11 prefecture level cities in Hebei Province in 2000, 2005, 2010, 2015.
(XLS)

S2 Table Hourly PM_2.5_ concentration data in 2015. Hourly PM_2.5_ concentration data of 11 prefecture level cities in Hebei Province in 2015.
(XLS)

S1 Fig PM_2.5_ raster data of Hebei Province in 2000. The figure shows the PM_2.5_ concentration grid data (0.01°×0.01°) of Hebei Province in 2000.
(TIF)

S2 Fig PM_2.5_ raster data of Hebei Province in 2005. The figure shows the PM_2.5_ concentration grid data (0.01°×0.01°) of Hebei Province in 2005.
(TIF)

S3 Fig PM_2.5_ raster data of Hebei Province in 2010. The figure shows the PM_2.5_ concentration grid data (0.01°×0.01°) of Hebei Province in 2010.
(TIF)

S4 Fig PM_2.5_ raster data of Hebei Province in 2015. The figure shows the PM_2.5_ concentration grid data (0.01°×0.01°) of Hebei Province in 2015.
(TIF)
