# Supplementary figures and images for: Study on the relationship between PM2.5 concentration and intensive land use in Hebei Province based on a spatial regression model
Source: PLoS One. 2020 Sep 18;15(9):e0238547. doi: 10.1371/journal.pone.0238547 (PMC7500636; doi:10.1371/journal.pone.0238547)

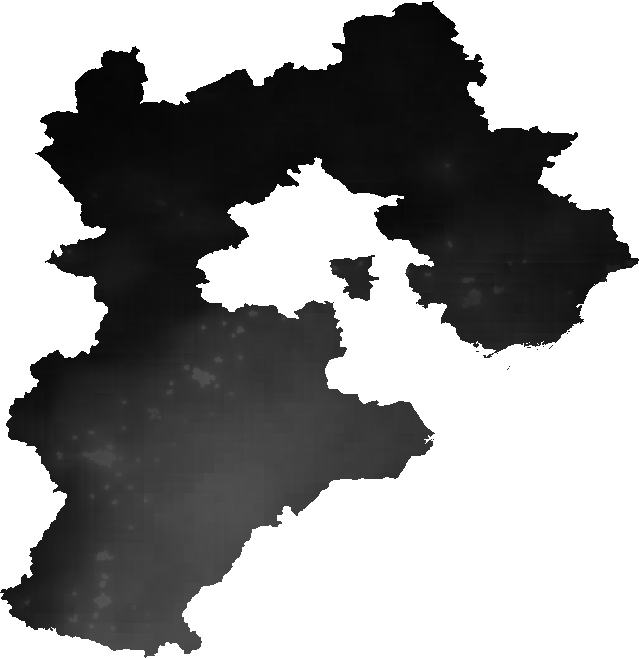

Supplement: S1 Fig — The figure shows the PM2.5 concentration grid data (0.01°×0.01°) of Hebei Province in 2000. (TIF) [file pone.0238547.s003.tif]

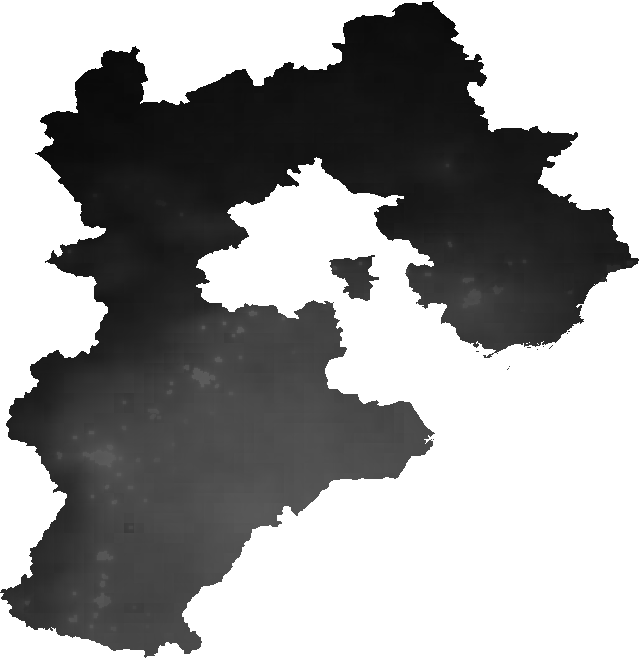

Supplement: S2 Fig — The figure shows the PM2.5 concentration grid data (0.01°×0.01°) of Hebei Province in 2005. (TIF) [file pone.0238547.s004.tif]

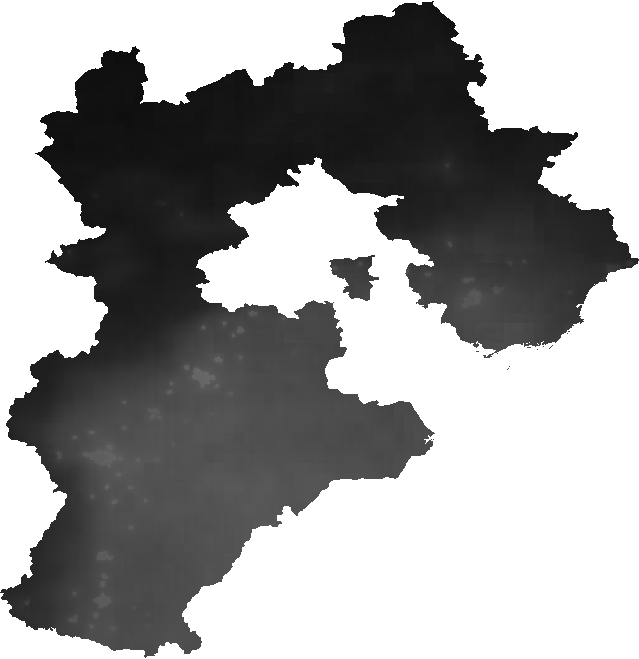

Supplement: S3 Fig — The figure shows the PM2.5 concentration grid data (0.01°×0.01°) of Hebei Province in 2010. (TIF) [file pone.0238547.s005.tif]

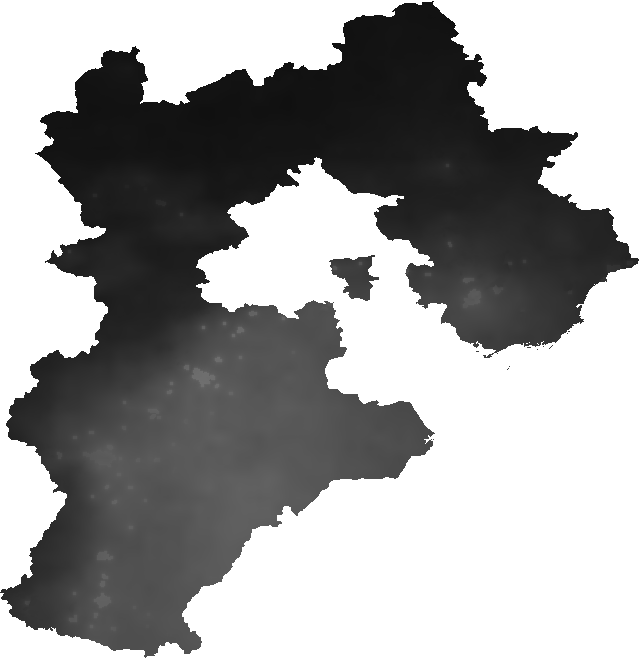

Supplement: S4 Fig — The figure shows the PM2.5 concentration grid data (0.01°×0.01°) of Hebei Province in 2015. (TIF) [file pone.0238547.s006.tif]
